# Adolescent Obesity: Diet Quality, Psychosocial Health, and Cardiometabolic Risk Factors

**DOI:** 10.3390/nu12010043

**Published:** 2019-12-23

**Authors:** Lyndsey D. Ruiz, Michelle L. Zuelch, Sarah M. Dimitratos, Rachel E. Scherr

**Affiliations:** 1 Department of Nutrition, University of California, Davis, Davis, CA 95616, USA; ldruiz@ucdavis.edu (L.D.R.); mzuelch@ucdavis.edu (M.L.Z.); smdimitratos@ucdavis.edu (S.M.D.); 2 Center for Nutrition in Schools, University of California, Davis, Davis, CA 95616, USA; 3 Stress Biology and Human Nutrition Research Lab, Obesity and Metabolism Research Unit, USDA Western Human Nutrition Research Center, Davis, CA 95616, USA

**Keywords:** adolescent, obesity, severe obesity, diet quality, psychosocial health, stress, cardiometabolic risk, food literacy

## Abstract

Obesity is a multifaceted chronic condition with several contributing causes, including biological risk factors, socioeconomic status, health literacy, and numerous environmental influences. Of particular concern are the increasing rates of obesity in children and adolescents, as rates of obesity in youth in the United States have tripled within the last three decades. Youth from historically disadvantaged backgrounds tend to have higher rates of obesity compared to other groups. Adolescents often do not meet intake recommendations for certain food groups and nutrients, which may contribute to a heightened risk of obesity. With obesity disproportionately affecting adolescents (ages 12–19 years), negative effects of excess adiposity may be particularly salient during this critical period of development. The presentation of chronic cardiometabolic disease symptoms typically observed in adults, such as hypertension, hyperglycemia, dyslipidemia, and inflammation, are becoming increasingly common in adolescents with obesity. Additionally, there is dynamic interplay between obesity and psychosocial health, as adolescents with obesity may have increased levels of stress, depressive symptoms, and reduced resilience. To reduce and prevent adolescent obesity, the implementation of theory-driven multicomponent school- and community-based interventions have been suggested. These interventions promote knowledge and self-efficacy for healthful practices that have the potential to progress to sustained behavior change.

## 1. Introduction

Adolescence is a critical period of development, defined by navigating challenging social circumstances and cementing identity as youth transition into emerging adulthood [1]. Adolescence is also a time of immense growth—second only to the first year of life—and as such, nutrient requirements increase substantially [2]. Data from the National Health and Nutrition Examination Survey (NHANES), which surveys representative samples from varying age groups of the United States, are collected every fiscal year, referred to as a cycle, on an array of health-related topics including overweight and obesity status [3]. In youth, overweight and obesity status is commonly classified by age- and sex-specific body mass index (BMI) percentiles, determined by growth charts developed by the Centers for Disease Control and Prevention using historical data from national surveys [4]. Measurement at or above the 85th percentile and below the 95th percentile on the age- and sex-specific growth charts indicates overweight status, while measurement at or above the 95th percentile indicates obesity [5,6]. Severe obesity in children and adolescents is defined by a BMI percentile at or above 120% of the 95th percentile [6]. As identified by NHANES, rates of childhood and adolescent obesity have more than tripled since the 1970s and severe obesity rates have more than quintupled within the same timeframe [7,8]. Data collected from the 2015–2016 NHANES cycle indicated that 18.5% of youth aged 2–19 years in the United States were obese, of which 5.6% were classified as severely obese [7]. Adolescents aged 12–19 years, the age range utilized throughout this review to define adolescence, had the highest prevalence of obesity at 20.6%, compared to 18.4% for youth aged 6–11 years and 13.9% for children aged 2–5 years [7]. Even more concerningly, youth from different ethnic groups are disproportionally obese, with Mexican American, Hispanic, and non-Hispanic black youth having above average prevalence of obesity [7,8]. 

Consistently, overweight or obese children and adolescents are more likely to have elevated BMIs as adults [6,9,10]. Adolescents with BMIs above the 85th percentile are more likely to be obese by age 35 than their normal weight counterparts [6]. The probability of being overweight or obese as an adult increases with both youth BMI percentile and age, with obese adolescents being at the highest risk for obesity during adulthood [6,9,11,12]. In particular, youth who are obese during their teenage years have an over 90% likelihood of being overweight or obese at 35 years [9]. A study that combined several national datasets to model obesity trajectories from childhood through to middle age found that overweight or obesity at age 18 increased the risk of being obese in adulthood and that risk for adult obesity was more accurately assessed in adolescents rather than younger age groups [12]. 

Similar to adults, obesity in adolescents impacts all major organ systems and often contributes to morbidity [13,14]. Adolescent obesity promotes inflammation and increases the risk of chronic disease development into and throughout adulthood [15]. Compared to those who are of normal weight, adolescents who are obese are at increased risk for adverse health effects, including cardiovascular risk factors such as hypertension, dyslipidemia, and endothelial dysfunction [16,17,18,19,20,21], and metabolic risk factors including insulin resistance and hyperglycemia [16,22,23,24,25,26]. These risk factors persist throughout adolescence and into adulthood [14,16,21,25,26,27,28,29,30,31]. The diminished cardiometabolic health status that often digresses throughout adolescence is associated with the adoption of poor dietary and physical activity behaviors [27].

## 2. Factors Contributing to Adolescent Obesity

### 2.1. Diet Quality

Diet quality can be measured to better understand overall eating patterns. One common method for assessing diet quality is through the generation of Healthy Eating Index (HEI) scores, from either food frequency questionnaires or 24 h recalls [32]. Currently NHANES utilizes two 24 h recalls to collect dietary information; however, prior to 2002, only one 24 h recall was collected [33,34]. The HEI allows for the assessment of overall diet quality as well as individual dietary components, including those recommended to be limited in the diet [35,36]. Higher HEI scores indicate an eating pattern more congruent with dietary recommendations than lower scores, and are based on a coordinating edition of the Dietary Guidelines for Americans, which is updated in accordance every five years [32,35]. The current method of the HEI was first created to coincide with the Dietary Guidelines for Americans, 2005 and MyPyramid recommendations [37]. This version of the index assessed diet quality through adequacy, or moderation, of 12 components as a ratio of overall energy intake [37]. Many of these components were carried forward into the subsequent version of the HEI in 2010, although milk, meats and beans, and calories from solid fats, alcoholic beverages, and added sugars were renamed to dairy, total protein foods, and empty calories, respectively [35]. The remaining components were modified in this update to better reflect updated dietary recommendations, including the inclusion of seafood and plant proteins and adding refined grains as a component to limit [35]. For the most recent update, HEI-2015, the only component change was the splitting of empty calories into saturated fat and added sugars [36]. Therefore, HEI-2015 is a composite score of 13 components to assess how well individuals meet key recommendations outlined in the 2015–2020 Dietary Guidelines for Americans [32,36,38]. As with previous versions, an overall HEI-2015 score ranges from 0 to 100, with 100 representing an eating pattern exactly aligned with recommendations [32,36].

Youth in the United States do not meet dietary recommendations and adolescent diet quality is of particular concern. An expansive analysis of NHANES data covering seven cycles, from 1999 to 2012, included over 17,000 adolescents aged 12–18 years, out of a total of over 38,000 youth aged 2–18 years [33]. This study assessed adolescent diet quality utilizing HEI-2010, as well as analyzing trends over time [33]. Consistent with other analyses [33,39,40,41,42], overall diet quality was shown to decrease with age as adolescents persistently had significantly lower overall HEI scores compared to younger youth [33]. Furthermore, results indicated that adolescents 12–18 years had an average overall HEI score of 48.4 out of 100 in the 2011–2012 NAHNES cycle, which was a significant increase from the average overall score of 40.4 observed during the 1999–2000 cycle [33]. On trend with overall diet quality improving over time [33], the overall HEI-2015 score for adolescents 12–18 years was 52.0 in a more recent analysis [42]. Although overall diet quality scores have significantly improved over time for adolescents, the current scores are still considered low [33,42].

Many countries, including the United States, have food-based dietary guidelines that include recommendations for food group consumption [43]. The majority of countries that have food-based dietary guidelines use five food groups, including starchy staples (grains for MyPlate in the United States), fruits, vegetables, dairy foods, and protein foods. While these guidelines may be more understandable to the general public, they do not provide recommendations for the consumption of specific nutrients. Most recent analyses of dietary data collected from NHANES show that, in general, adolescents are able to meet recommendations for protein including having better consumption of seafood and plant sources of protein compared to younger age groups [42]. However, these analyses also suggest that adolescents are not meeting recommendations for fatty acids [42]. The HEI-2015 scoring for fatty acids is based on a ratio of polyunsaturated and monounsaturated fatty acid relative to saturated fatty acid intake [36]. With an average score of only 3.7 out of 10, it is likely that adolescents generally consume higher levels of saturated fatty acids in comparison to unsaturated fatty acids [42]. Additionally, the overall poor diet quality of adolescents is driven by the inadequate consumption of components considered more healthful, such as fruits, vegetables, and whole grains [39,40,41,42,44]. Analyses suggest that adolescents only consume about half the recommendations for fruits and vegetables [39,41,42] and with an average HEI score of 1.32 out of a possible 10, were consuming below the whole grains recommendation in one analysis [39]. The inadequate intake of these food groups perpetuated dietary fiber to be recognized as a nutrient of concern in 2015–2020 Dietary Guidelines for Americans [45]. Another recommendation outlined in the 2015–2020 Dietary Guidelines for Americans is to shift away from consuming added sugars [45]. Before becoming a singular category in HEI-2015 [36], added sugars were included in the HEI-2010 “Empty Calories” component, along with solid fats and alcohol, and are calculated negatively into the overall score [35]. While data from NHANES 2005–2010 suggested that adolescents had high consumption of empty calories [39], a separate analysis utilizing data from NHANES 1999–2012 showed a substantial decrease in empty calorie consumption over time [33]. Although this trend was an improvement, empty calorie consumption in adolescents still exceeded recommendations in both studies [33,39] and adolescents were only meeting about half the recommendation for reducing added sugar consumption in a more recent study using HEI-2015 [42].

### 2.2. Socioecological Influences

In addition to developmental changes, adolescence is a period of social change, with adolescents progressing toward increased autonomy, and perhaps may result in the establishment of dietary habits [2,46]. The Social–Ecological Model describes that food choices can be influenced from several different levels, spanning from intrapersonal factors to policy and systems [47,48]. These sectors of influence can have differential effects on an individual’s risk for overweight or obesity.

Ethnicity and socioeconomic status (SES) are two factors that are associated with youth obesity rates. The prevalence of obesity is higher in Hispanic and non-Hispanic black youth compared to non-Hispanic white youth within the same age group [49]. In 2016, the prevalence of obesity for Hispanic and non-Hispanic black adolescents aged 12–19 years were 25.9% and 25.0%, respectively, which was substantially higher than the 17.2% observed in non-Hispanic white adolescents [49]. The prevalence of severe obesity was also highest among these groups, with 11.6% of Hispanic adolescents and 11.5% of non-Hispanic black adolescents being considered severely obese, compared to only 6.7% of non-Hispanic white adolescents [49]. In line with these values, data collected through NHANES suggest that non-Hispanic black adolescents typically have the lowest overall diet quality scores compared to other youth [33,41,42]. However, Mexican-American and Hispanic adolescents tend to have the highest overall diet quality and component scores compared to other groups [33,41,42], which is surprising given the high prevalence of overweight and obesity observed in Hispanic youth [7]. Similar results were found in a study assessing the diet quality of high school students utilizing HEI-2010, with Hispanic students having higher overall HEI scores compared to non-Hispanic white youth [50]. One potential explanation for this observation is the lack of physical activity opportunities for adolescents from some ethnic/racial minority groups and communities of lower SES. Analyses of NHAHES 2007–2016 data showed that adolescents from low-income families participated in less physical activity than more affluent adolescents [51]. This association may be the result of reduced access to parks, playgrounds, and exercise facilities, which is more prevalent in less advantaged communities [52]; a problem that is even more prevalent in communities where the population is predominantly of an ethnic/racial minority group [53]. A nationally representative study found that neighborhoods primarily comprising ethnic/racial minority and low SES groups were half as likely to have access to a physical activity facility on their block [53]. This is a substantial disadvantage given that the assessment also found that access to one of these facilities significantly decreases the odds of adolescent overweight [53].

An analysis that included 10 years of NHANES data sought to better characterize the role SES plays in modifying diet quality of Mexican-origin youth [54]. For this study, high or low SES was estimated with consideration for education and income-to-poverty-ratio [54]. As in other analyses of NHANES data [33,41,42], Mexican-origin youth of the same generation as non-Hispanic white youth had higher overall diet quality, as determined by HEI-2010 scores [54]. Interestingly, the average HEI score for overall diet was significantly lower in third-generation Mexican-origin youth from low SES families compared to first and second generations [54]. This decrease in overall diet quality as generation progressed was perceived to be from acculturation and the increased consumption of empty calories, as is more customary in a typical American diet [54]. The trend in later generations having poorer diet quality was attenuated by SES as no significant differences in diet quality were observed between generations from high SES families [54]. Unlike the association found with Mexican-origin youth, overall diet quality scores from NHANES data have either shown no difference between the highest and lowest income youth [41] or were occasionally significantly associated with income level, but the direction of this association was not consistent over time [33]. Despite this, lower income households tend to have a higher prevalence of obesity than higher income households [55]. Similarly, there is, generally, an inverse relationship between head of household education attainment and youth obesity [55]. In 2016, youth obesity prevalence was highest for those whose head of household did not receive a high school diploma [49].

Youth from lower SES families are also more likely to experience food insecurity [56,57,58]. Food security can be categorized into one of four ranges: very low, low, marginal, and high food security [59]. Classification into one of these ranges is determined by how often a family or individual experiences distress involving food selection or alters eating patterns due to insufficient resources to obtain food [57,59]. The United States Department of Agriculture monitors food insecurity rates utilizing an annual survey. Most recent estimates have shown a continuous decline in the percentage of food insecure households since 2011 [57]. While low-income families and households with children, in particular non-Hispanic black and Hispanic households, remain at percentages above the national average [57], this shift in prevalence is promising given that household food insecurity is related to overweight and obesity in youth [56,58,60,61].

Adolescence is marked by increased autonomy and a transition from spending the majority of time with parents to away from home with peers [46,62]. While parents still provide guidance on certain matters, peers assert more influence on superficial concerns, especially as adolescents enter teenage years [62]. This influence in regard to eating behaviors may be perpetuated by a desire to fit into a particular peer group, among other complex factors [63]. Peer influence is evident in adolescent selection and consumption of food [63], with mixed observations on whether the tendency is toward encouragement or discouragement of consuming healthy foods [63]. Peers, especially friends, can have a beneficial effect on adolescent eating patterns. One study found that adolescent diet quality scores were positively related to healthy food choices made by peers [46]. Another study found that healthful aspects of best friends’ eating patterns can be influential for adolescents and result in consumption of significantly more vegetables, whole grains, and dairy [64]. While statistically significant, the increases observed in this analysis were not substantial, with adolescents consuming an additional 0.09, 0.14, and 0.08 servings of vegetables, whole grains, and dairy, respectively [64]. In practice, the 0.08 serving increase in dairy would be roughly equivalent to 0.5 ounces of fluid dairy or about one tablespoon of milk. A cross-sectional study assessing youth and adolescent diet quality, observed no relationship between overall HEI-2010 score and friend support for eating healthy or unhealthy foods [65].

Despite increased autonomy, parents still play a role in shaping adolescent eating. Parents influence adolescent eating patterns through food procurement and by modeling and supporting healthy eating behaviors [46,66,67,68,69]. In the cross-sectional analysis mentioned previously, parental offering of food considered unhealthy was associated with decreased diet quality scores [65]. However, 40% of the sample also indicated that their parents rarely or never offered unhealthy foods, thus modifying their availability and accessibility [65]. If high-fat foods and sweets are not being offered, then consumption may be limited allowing for higher adolescent diet quality. Furthermore, the availability of fruits and vegetables in the home is correlated with adolescent fruit and vegetable consumption [67,70].

## 3. Adolescent Stress and Adiposity

### 3.1. Physiological Stress

Adolescence is known to be a stressful developmental period, and emerging research supports the need to address psychosocial stress as a factor in obesity prevention and management [71,72,73,74]. The psychosocial stress arising from poor body image and social ostracization, especially associated with adolescent obesity, may further promote stress and corresponding health-compromising coping mechanisms [75]. Stress is broadly defined as the body’s response to a real or perceived threat beyond the ability to cope [76]. A perceived threat activates the neuroendocrine hypothalamic-pituitary-adrenal (HPA) axis, ultimately resulting in the secretion of cortisol from the fasciculata of the adrenal cortex [77]. Cortisol binds to receptors found in the peripheral and central nervous system, where its objective is to mobilize and redistribute energy stores to maintain homeostasis and minimize incurred damage to the individual until the threatening stimulus has passed [78]. Outcomes of chronic HPA-axis activation include effects on gluconeogenesis and glycogenolysis [79], lipolysis [80], insulin resistance [81,82], and compromised reproductive functions [83].

Cortisol is essential for organism survival [84]. However, the effects of chronic stress are systemically deleterious, as glucocorticoid receptors are ubiquitously spread throughout body tissues, such that nearly every organ system is affected [85]. Under chronic stress, the characteristic negative-feedback nature of the HPA-axis may become dysfunctional, which increases the risk of developing a host of metabolic and affective disorders [85]. Prolonged stress contributes to allostatic load, where the body develops new “set points” including, but not limited to, higher blood glucose, stress sensitivity, and reactivity [86]. Chronic psychosocial stress promotes metabolic derangement including adiposity, as well as abnormal eating behaviors including over- or under-eating, and preferentially selecting highly palatable foods [87]. Furthermore, prolonged stress also confers increased risk for developing numerous chronic diseases, including metabolic syndrome [88], diabetes mellitus [89], cardiovascular disease [90], obesity [71], and mental health disorders [91].

### 3.2. Adolescent Stress

Adolescents are especially vulnerable to the negative effects of stress, at least partially due to the sensitization of the HPA-axis that occurs during this period [92]. Adolescence is a developmental period marked by heightened stress reactivity and sensitivity, increased emotionality, and increased incidence of both risk-taking and harm-avoidant behaviors [93]. Adolescents typically experience heightened stress sensitivity and prolonged reactivity in a sex-dependent manner, with basal and stress-responsive cortisol typically higher in females [94]. Many of the affective and behavioral signatures typical of adolescence can be explained by rapid gonadal hormone development and non-linear neurodevelopment [95]. In adolescents, limbic brain regions involved with motivation, instant gratification, and reward develop much more rapidly than do cortical regions involved in inhibitory control [93]. Thus, limbic brain circuitry is more likely to predominate over less mature cortical regions during emotionally salient contexts [96]. The effects of stress on metabolism and food choice plus the psychosocial stress experienced by adolescents with obesity are critical points for consideration.

## 4. Stress-Motivated Eating Behavior

Both animal and human studies have demonstrated that the majority of individuals preferentially select highly palatable foods when stressed, whether-or-not they exceed their caloric requirements [87,97]. This once conferred evolutionary advantage, as additional calories increased the likelihood of escaping from or fighting—and thus surviving—what were historically acute physical threats [98]. Modern stress is largely chronic and psychogenic in nature rather than physical [98]. These chronic stressors, coupled with a more sedentary modern lifestyle, result in an evolutionary mismatch; the body employs conserved response mechanisms to psychosocial stress, which involve increased drive to seek out palatable foods meant to aid in fighting or fleeing a threatening situation [76]. Repeated exposure to psychosocial stressors, and the resultant consumption of such highly palatable foods in our modern environment may, ultimately, increase the risk of developing overweight and obesity.

Adolescents are at increased risk of partaking in unhealthy behaviors, especially in emotionally salient contexts [95]. Maturation in brain regions involved in reward seeking may underpin the drive for palatable food consumption in adolescence [99]. In fact, the repeated consumption of palatable foods in this critical window of neurodevelopment may derail normal maturation processes, thus predisposing the adolescent brain to abnormal eating behaviors [99]. Palatable foods eaten under stress are typified by sweet taste and tend to be foods high in rapidly digesting, simple carbohydrates [100]. The physiologic signals that arise from consuming palatable foods rich in simple carbohydrates orchestrate cognitive, metabolic, and behavioral responses to stress, which, over time, may increase obesity risk [101,102]. Importantly, sweet taste is instantly rewarding, and may promote reinforcement learning—even in the absence of post-prandial metabolic signals, which can also contribute to overconsumption and obesity [103]. This was exemplified when rats given oral administration of sucrose solution demonstrate reduced stress responses, whereas intragastric gavage of sucrose had no such effect [104]. In humans, this attenuation of stress in response to consuming palatable foods high in simple carbohydrates has been shown when exogenous carbohydrate consumption before a combined mental and physical stress challenge mitigated effects of stress [105].

### Metabolic Effects of Palatable Food Consumption

Stress-related emotional eating in the absence of hunger involves the motivation and reward-associated brain networks that override homeostatic feeding cues originating from the hypothalamus [106]. The post-ingestive metabolic signals arising from continually exceeding caloric requirements for weight maintenance promote increased energy storage and reduced expenditure, and these effects are exacerbated under stress [107]. Postprandial effects of consuming palatable foods include blood glucose elevation, which is met by an increase in insulin secretion [108]. Effects of insulin in tandem with the effects of cortisol on disruption of glucose and insulin homeostasis, further promote energy storage, especially in the visceral region [109]. In addition to promoting glucose homeostasis, insulin also interacts with neuropeptides to increase energy expenditure and reduce food intake in the absence of stress [110]. The neuroendocrine axes orchestrating stress and energy balance overlap, with notable neuropeptides and hormones involved in energy balance also influencing stress regulation [111].

Leptin is an adipocyte-derived hormone with anorectic effects and has been shown to dampen HPA activity associated with chronic stress [112]. Leptin has both central and peripheral targets, where combined effects with insulin and other anorexigenic hormones result in, but are not limited to, alterations in food intake, glucose and lipid metabolism, pancreatic islet B-cell secretion, reproductive function, immunity, and energy expenditure [113,114]. In the fed state, centrally acting leptin is secreted from the arcuate nucleus of the hypothalamus, then activates neurons associated with increased satiety and energy expenditure, and inhibits neurons associated with increased food intake and weight gain [115]. Circulating leptin concentrations are often high in individuals with obesity, thus suggesting a state of leptin resistance [112,116,117]. Whereas leptin deficiency can be corrected with exogenous recombinant leptin administration, leptin resistance is not attenuated with the introduction of additional hormones [118]. With respect to stress, one study showed that a seven-day glucocorticoid treatment intervention resulted in increased food intake despite increased serum leptin levels [119]. Conversely, another study demonstrated that high glucocorticoids after a social stressor were associated with transient increases in plasma leptin, thus resulting in temporarily suppressed appetite and food consumption under stress [120]. Hypercorticism is oftentimes observed in obesity, and glucocorticoids are known to restrain the effects of leptin [121]. Thus, psychogenic stress promotes metabolic dysregulation directly and indirectly through its influence on hormones and neuropeptides involved with energy balance.

## 5. Obesity-Associated Psychogenic Stress in Adolescents

Obesity can be a stressful state due to weight stigma [122] and adolescents who experience stress related to social ostracization are more likely to rely on food-related coping mechanisms [123]. This behavior is immediately rewarding and may contribute to temporary solace and improved mood [124], however, repeated intake in excess of caloric needs will result in weight gain, thus perpetuating the cycle [125]. Psychogenic stress as a result of weight stigma may contribute to disordered eating habits in adolescents [126]. A prospective cohort study collected 10 waves of data from 1420 participants and found that victims of bullying in childhood and adolescence had an increased likelihood of developing anorexia nervosa and bulimia [127]. Furthermore, adolescents experiencing weight-related stigma are at increased risk of engaging in secretive eating, characterized by eating in solitude to avoid being seen by others [128]. Secretive eating is correlated with binge eating and the onset of other eating disorders, and may be related to depression and poor body image [128]. In a cross-sectional study examining 577 youth, those endorsing secretive eating experienced greater eating-related psychopathology [128]. Additionally, it was found that adolescents experience more dietary restraint and purging than younger youth [128].

Sex differences, personality types, cultural and familial normative beliefs, self-worth, and learned coping mechanisms all inform the extent to which an individual internalizes and copes with psychosocial stress [129]. For example, neuroticism partially accounted for associations between depression and chronic life stress in 603 adolescents in a study exploring risk factors for emotional disorders [130]. Furthermore, depression was associated with chronic life stress in females only, and low extraversion partially accounted for associations between social phobia and chronic life stress [130]. With regard to sex differences, female sex hormones contribute to higher stress sensitivity and sustained stress responses [94]. Adolescents are at an increased risk for dieting with the goal of weight loss [131], and those who experience personal factors such as weight concern, body dysmorphia, and depression are more likely to develop disordered eating behaviors 10 years later [132]. One study found that body image dissatisfaction in adolescent females was associated with self-esteem [133]. Females are also at higher risk of developing eating disorders [134]. Finally, restrained eaters, those who consciously elect to restrict intake of food quantity or food types [135], are at higher risk of emotional eating compared to unrestrained eaters [136].

## 6. Obesity and Cardiometabolic Disease in Adolescents

### 6.1. Cardiovascular Disease

Cardiovascular disease (CVD) is the number one cause of death in the United States [137]. By the year 2030, the percentage of the population suffering from CVD is projected to approach 44% [138]. Although CVD is generally perceived as a disease of adulthood, studies suggest that atherosclerosis often begins in childhood or adolescence [26,139,140]. Cardiovascular risk develops as a culmination of the atherogenic process over the lifespan [20,26,28,141]. Progression of atherosclerosis is related to the number and intensity of cardiovascular risk factors, which develop in childhood and track into adulthood [21,26,142] and may be independent of adult weight [143]. It is estimated that 70% of obese children and adolescents ages 5–17 years have at least one cardiovascular risk factor [144]. Risk factors—for example, hypertension and dyslipidemia—are directly, positively associated with the presence and severity of early atherosclerotic lesions in adolescents and young adults [20,140]. Obese children have been observed to have significantly impaired arterial elasticity and endothelial function [21]. In addition, obesity in youth is associated with increased cardiac mass and intima-media thickness in adulthood [145,146,147]. Out of the cardiovascular risk factors, obesity is the most predictive of future disease [22,142] and adolescent obesity is projected to yield an increase in coronary heart disease in adulthood [26].

The Bogalusa Heart Study, a long-term epidemiologic study of cardiovascular disease risk factors beginning in childhood, assessed cardiovascular risk factors, including serum lipid concentration, blood pressure, and BMI, in children and adolescents, following them from youth into adulthood [22,141,142]. Findings suggest that intensity of cardiovascular risk in youth predicts subclinical atherosclerosis and adult morbidity and mortality [27,142]. The Pathobiological Determinations of Atherosclerosis in Youth Study, another large-scale study of atherosclerosis in adolescents and young adults (15–34 years) also assessed the presence and extent of early atherosclerotic lesions in relation to cardiovascular risk factors in subjects who underwent autopsy [148]. Their results were in agreement with those from the Bogalusa Heart Study; intimal lesions were present in the aorta of all subjects aged 15–19 years and severity increased with age [148]. Other studies report that specifically an android fat distribution, or central adiposity, is correlated with dyslipidemia and arterial stiffness in youth [20,21]. Tounian et al. [21] suggested that android fat distribution, dyslipidemia, and insulin resistance may be primary contributors to these vascular impairments. Obese youth had significantly higher levels for each of these parameters [21], which aligns with several other studies examining lipid profile, blood pressure, and glucose and insulin concentrations in obese youth [144,149,150,151].

Severity of obesity is also relevant [14,152]. In a large-scale, cross-sectional study utilizing data from NHANES 1999–2012, researchers observed that all cardiometabolic risk factors were elevated as severity of obesity increased in adolescents [153]. When controlling for age, race/ethnicity, and sex, greater severity of obesity yielded increased risk of dyslipidemia, hypertension, and elevated glycated hemoglobin level [14].

### 6.2. Type 2 Diabetes Mellitus

Similar to CVD, insulin resistance and type 2 diabetes mellitus (DM) are obesity-related complications previously thought to develop in adulthood that are becoming increasingly more prevalent in younger populations [25,154,155]. As with adults, central adiposity in youth is associated with insulin resistance [21]. The first metabolic abnormality seen in obese youth is hyperinsulinemia [156]. The decrease in insulin sensitivity that occurs with puberty further compounds insulin resistance in obese adolescents [23]. In addition to the inflammatory response, adiponectin is considered to partially explain the relationship between obesity and type 2 DM [157]. Due to its negative association with insulin resistance [158], adiponectin has been considered an insulin-sensitizing adipokine [157,159] and is inversely related to adiposity [150,160]. Obesity is also strongly, negatively correlated with adiponectin level in adolescents, as well as in children and adults [161,162]. Lower levels of adiponectin are associated with increased levels of insulin resistance in obese adolescents [163] such that most youth with insulin resistance are overweight or obese [158]. In addition to obesity and insulin resistance, low adiponectin levels in youth are also associated with hypertension and dyslipidemia and may therefore predict the clustering of these symptoms of metabolic syndrome [24,164]. The presence of these risk factors in obese children and adolescents compounds the risk for the development of subsequent type 2 DM and CVD in youth [150].

### 6.3. Compound Risk: Obesity, Diabetes, and Cardiovascular Disease

The diagnosis of DM is an established risk factor for vascular disease and the early development of CVD [26]. The metabolic abnormalities in energy utilization that are associated with DM cause diabetic dyslipidemia [165]. Also, the chronic hyperglycemia often seen in combination with obesity results in damage to the vasculature [166]. For this reason, adolescents with obesity are at significantly increased risk for accelerated atherosclerosis [26]. Children with the described cluster of metabolic abnormalities were more likely to have type 2 DM and clinical cardiovascular events after a follow-up of 25 years [30,31]. Even in absence of the metabolic abnormalities, there is a strong association between obesity in adolescence and subsequent development of the metabolic syndrome cluster in adulthood [26], which may be attributed to obesity-induced chronic inflammation [167]. Pro-inflammatory adipokines—for example, leptin—have been implicated in the development of both obesity-related type 2 DM and CVD [154]. Obesity-induced insulin resistance is also associated with increased carotid intima-media thickness [168] and endothelial dysfunction in obese adolescents [169,170].

## 7. Intervention Opportunities

Without intervention, it is projected that most youth will be overweight or obese and likely suffering from chronic diseases in adulthood given current expected trajectories [11,12,25]. Diet and lifestyle modification in adolescence or earlier is essential in the prevention of the development of chronic diseases in adulthood. The concept that youth are in the subclinical stages of cardiometabolic disease, which may be exacerbated by stress, emphasizes the need for early intervention [14,18,27,150,171,172,173]. The abnormal accumulation of lipids in the vascular wall is a reversible stage in the atherogenic process [26], making the early stages of atherosclerosis, which often appear in youth, an ideal opportunity for intervention. Early identification and intervention may attenuate clinical manifestation and improve long-term health outcomes [26]. Analyses of results from several prospective longitudinal cohort studies found that risk for hypertension, dyslipidemia, atherosclerosis, and type 2 DM in obese youth who became non-obese by adulthood were similar to those who were never obese [142,147,174,175]. These findings suggest that weight management in youth, adolescence, and young adulthood may at least partially diminish cardiometabolic risk in adulthood [29]. Weight loss coupled with lifestyle modifications, including stress reduction, increased physical activity, and improvements in diet, is often sufficient to improve insulin sensitivity and can thereby assist in the prevention or control of type 2 DM without the need for exogenous insulin administration [154,176,177].

According to the United States Burden of Disease Collaborators, the primary risk factor related to disease burden was found to be suboptimal diet [178]. It has been recommended that interventions geared toward improving adolescent dietary behaviors are thoroughly planned in advance to ensure that they are designed, implemented, and monitored appropriately for the targeted population [179]. In designing interventions, the most successful have been developed in line with a theoretical framework, most commonly the Social Cognitive Theory (SCT) [179]. The SCT is utilized in nutrition interventions due to its consideration for improvement of individual factors, such as self-efficacy and knowledge, as well as environmental factors, when facilitating behavior change [180]. Utilizing SCT as the guiding theoretical framework also aids in designing behaviorally-focused interventions that modify the environment while also being developmentally appropriate for the intended participants, which have also been implicated as elements of successful nutrition interventions [179].

Nutrition interventions for adolescents are frequently implemented through comprehensive school-based interventions and multicomponent programming, as recommended by the Academy of Nutrition and Dietetics, Society for Nutrition Education and Behavior, and School Nutrition Association [181]. Multicomponent school-based interventions have shown promise for improving dietary intake and health status of children and adolescents [182]. Multicomponent programs commonly include nutrition education implemented in the classroom; modifications to school policies and the food environment; and methods for parental involvement [182].

Further recommendations include adjustments to the school environment in order to facilitate acquisition of healthful behaviors and the promotion of evidence-based nutrition education that includes opportunities for youth to grow and prepare food [181]. Programs that incorporate garden and cooking components are important given their potential for translatable and long-term effects. Gardening experience during childhood is valuable as it has been associated with significantly higher fruit and vegetable consumption during late adolescence compared to older adolescents with no prior gardening experience [183]. Furthermore, frequent gardening is beneficial in that gardening weekly or even monthly has been associated with high fruit and vegetable consumption compared to infrequent or no gardening [183]. A recent survey of high school students found that adolescents who had a home garden or experience with community gardening or farming were significantly more likely than others without experience to try new fruits and vegetables [184]. Additionally, adolescents with a home garden were more likely to consume adequate amounts of vegetables [184]. As for inclusion of cooking, a review of programs that incorporate cooking found that participation in these programs has the potential to beneficially impact youth knowledge, skills, and behaviors related to nutrition in addition to cooking [185]. It has been found that participation in food preparation during adolescence was associated with a continuation of enjoyment and involvement in food preparation as an emerging adult [186].

In accordance with these recommendations, several recent interventions targeting youth dietary habits have included garden components [187,188,189,190,191,192,193] and cooking components [189,191,192,193,194,195]. While the age range in most of these studies included ages prior to adolescence [187,188,191,193,194,196], methods utilized and findings from these studies may have application for adolescents. Compared to controls, programs that included a gardening component resulted in greater willingness to try vegetables [187,188,197], preferences for vegetables [187,188,197], and reported vegetable consumption [187,190]. Similarly, participants in a nutrition program geared toward cooking had improvements in reported fruit and vegetable post-intervention consumption [194]. This program also resulted in increased reported nutrition knowledge, cooking self-efficacy, and cooking at home after completing the intervention [194]. Programs that included both gardening and cooking components observed that, compared to controls, participants had significantly higher fruit and vegetable [193] and nutrition knowledge [196]. Additionally, youth participating in the programs were significantly more likely correctly identify vegetables [196], consume fruits and vegetables daily [193], and be willing to try new foods [191].

The emerging concept of food literacy takes recommendations for the incorporation of growing and preparing food further. Broadly, food literacy is defined as the interconnection between the knowledge, skills, and behaviors necessary for procuring, planning, and preparing healthful food [198]. Food literacy is quite complex, encompassing several components including elements of nutrition, health, agriculture, food systems, food safety, and cooking [198,199,200]. Three reviews of adolescent food literacy programs have been conducted recently [201,202,203]. Two of these reviews highlight the need for a reliable and validated questionnaire to assess food literacy as a whole, given that none of the studies reviewed supplied such an assessment [201,203]. Varying interpretations of food literacy prior to establishment of a definition [198] limited the ability to develop an assessment to encompass the complexity of food literacy. With this and contrasting study designs, all three reviews noted difficulty in determining inclusionary criteria for articles and interpreting results [201,202,203]. Given the limitations of previous adolescent food literacy programs, Brooks and Begley compiled a list of recommendations for future programs, including the development of adaptable school-based food literacy programs for older adolescents [202].

## 8. Future Directions

Interventions are needed to aid in the reduction and prevention of adolescent obesity. As obesity is a complex health concern with numerous contributing factors, effective intervention strategies may require a multifactorial approach aimed at reducing stress and cardiometabolic risk factors while also empowering adolescents with the knowledge and skills necessary to make informed and healthful dietary choices. Some studies have suggested that mindfulness interventions for adolescents may be feasible for decreasing distracted eating [204] and reducing stress and depressive symptoms [205], which can aid in reducing and preventing obesity. Additionally, interventions that include exercise and calorie restriction components can be effective at reducing obesity and cardiometabolic risk factors [206,207]. These methods—in combination with multicomponent school-based interventions and skill-building health education programs, such as those that promote food literacy—warrant further research. However, a thorough review of the literature consistently demonstrates a gap with respect to adolescent food literacy education. Given that food literacy education is a comprehensive approach to target the upstream behaviors leading to obesity and related comorbidities, the timeliness of a program to combat this issue is critical. Aligning with the above recommendations [202], guided by SCT [180], and the definition established by Vidgen and Gallegos [198], a food literacy curriculum for high school-aged adolescents has been developed [208]. The curriculum, Teens CAN: Comprehensive Food Literacy in Cooking, Agriculture, and Nutrition (Teens CAN), includes experiential lessons within twelve modules that comprise opportunities to advance food literacy [208]. Teens CAN [208] will be incorporated into an existing multicomponent program, the Shaping Healthy Choices Program [189], to provide an intervention aimed at improving diet quality and the overall health status of children and adolescents. While this is one suggested approach, the ultimate goal is to mitigate the effects of childhood and adolescent obesity. Resources including time, money, and effort should be allocated toward this type of obesity prevention programming.
